# Highly efficient biodegradation of reactive blue 19 under the activation of tea residue by a newly screened mixed bacterial flora DDMY2†

**DOI:** 10.1039/c9ra04507d

**Published:** 2019-08-09

**Authors:** Xuehui Xie, Xiulin Zheng, Chengzhi Yu, Qingyun Zhang, Yiqin Wang, Junhao Cong, Na Liu, Zhenjiang He, Bo Yang, Jianshe Liu

**Affiliations:** State Environmental Protection Engineering Center for Pollution Treatment and Control in Textile Industry, College of Environmental Science and Engineering, Donghua University 2999# North Renmin Road, Songjiang District Shanghai 201620 China xiexuehui@dhu.edu.cn xiulinzxl@163.com hyywsdklts@163.com zqyfqyy@163.com 18321195601@163.com junhaocong@163.com yangbo@dhu.edu.cn liujianshe@dhu.edu.cn +86-21-67792522; Shanghai Institute of Pollution Control and Ecological Security Shanghai 200092 P. R. China; School of Environment and Surveying Engineering, Suzhou University Suzhou Anhui 234000 China liuna900301@163.com; School of Metallurgy and Environment, Central South University Changsha 410083 P. R. China hzjcsu@csu.edu.cn

## Abstract

In this study, a newly screened mixed bacterial flora DDMY2 had high decolorization capacity for anthraquinone dye reactive blue 19 (RB19) and the decolorization efficiency of 300 mg L^−1^ RB19 could reach up to 98% within 48 h in the presence of tea residue. Results indicated that RB19 could be efficiently decolorized by flora DDMY2 in wide ranges of pH values (5.0–9.0), temperatures (30–40 °C) and initial dye concentrations (50–500 mg L^−1^) under the activation of tea residue. Concentration of tea residue had been proved to significantly impact the decolorization performance. UV-vis spectrophotometry, Fourier transform infrared spectrometry and liquid chromatography/time-of-flight/mass spectrometry analysis showed three identified degradation products and the possible degradation pathway of RB19 was speculated. High-throughput sequencing analysis revealed the community structures of bacterial flora before and after domestication by tea residue. Based on the result, it was inferred that *unclassified*_*o*_*Pseudomonadales*, *Brevibacillus*, *Stenotrophomonas* and *Bordetella* activated by tea residue were responsible for the excellent decolorization performance. Results of this research deepen our understanding of the biodegradation process of anthraquinone dyes by bacterial flora and broaden the knowledge of utilizing tea residue as a bioactivator in biological treatment.

## Abstract 2

Tea residue promoted the decolorization of RB19 by activating flora DDMY2, revealing corresponding degradation pathways and functional genera in DDMY2.

## Graphical abstract

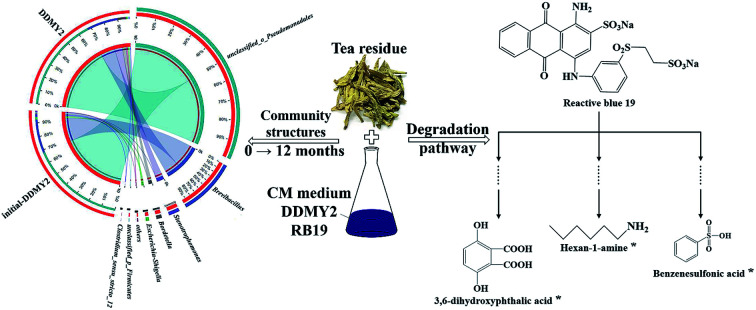

## 1. Introduction

Synthetic dyes are widely used in various industries, such as textiles, paper printing, cosmetics, plastics, food and leather.^1^ Among them, textile industries mainly have desizing, washing, bleaching, drying and other processes,^2^ which produce a large amount of effluent containing complex and refractory dyes. These dyes are discharged into water bodies, which can reduce the light penetration and oxygen transfer, resulting in changes to photosynthesis and aquatic ecosystems.^3^ Dyes are usually physically and chemically stable in the natural environment and therefore they are highly recalcitrant and harmful to living things.^4^

Given the refractory dye-containing wastewater, numerous physical and chemical treatment methods have positive effects on the removal of such wastewater. However, high cost and secondary pollution have greatly constrained these processes.^5^ Alternatively, biological treatment methods have been paid more attention, which have advantages of low cost, simple operation and environmental friendliness.^6,7^ At present, a vast literature on biological treatments using bacteria, fungi and algae have been reported to efficiently decolorize azo dyes.^8–10^ Unfortunately, the study on biodecolorization of anthraquinone dyes is still insufficient and the effect is undesirable. For instance, Holkar *et al.*^11^ isolated a strain of *Escherichia* sp. F NCIM 5545, which just decolorized 50 mg L^−1^ reactive blue 19.

Herein, tea residue as a domestic waste biomass, was employed to boost the decolorization performance of anthraquinone dye reactive blue 19 (RB19) by a newly screened mixed bacterial flora DDMY2. The impacts of several parameters (namely, pH, temperature, initial dye concentration and tea residue concentration) on decolorization were systematically investigated. UV-Vis spectrophotometry, Fourier transform infrared spectrometry (FTIR) and liquid chromatography/time-of-flight/mass spectrometry (LC-TOF-MS) were used to detect the biodegradation products of RB19. In addition, high-throughput sequencing revealed the microbial community structures of initial flora DDMY2 (not domesticated by tea residue) and flora DDMY2 (domesticated by tea residue for 12 months). The possible functional bacterial groups activated by tea residue were also speculated. These findings can provide new insights and extend the knowledge of biodegradation towards anthraquinone dyes. Moreover, this study can enlarge the utilization of waste biomass tea residue in industrial wastewater treatment.

## 2. Materials and methods

### 2.1. Chemicals and culture medium

Commercial grade reactive blue 19 (CAS No. 2580-78-1, MW 626.54, *λ*_max_ = 596 nm) was purchased from Sigma-Aldrich (USA). All other chemicals and reagents were of analytical grade, except for dichloromethane (HPLC level). The culture medium (CM medium) consisted of NH_4_Cl (0.20 g L^−1^), Na_2_SO_4_ (0.50 g L^−1^), KH_2_PO_4_ (2.66 g L^−1^) provided from Sinopharm Chemical Reagent (Shanghai) Co., Ltd. and yeast extract (3.00 g L^−1^) obtained from Sangon Biotech (Shanghai) Co., Ltd. Tea residue was made from West Lake Longjing tea (Hangzhou, China) which was repeatedly brewed at 80 °C until tea soup became colorless. The culture medium and tea residue were sterilized under the condition of 121 °C, 0.12 MPa for 20 min.

### 2.2. Screening of RB19 decolorizing bacterial flora DDMY2

RB19 decolorizing bacterial flora DDMY2 was screened from activated sludge taken from Songjiang sewage treatment plant (Songjiang, Shanghai, China). Through continuously increasing the dye concentration in the CM medium, activated sludge suspension (10%, v/v) was used to decolorize 300 mg L^−1^ RB19 until the decolorization rate reached more than 70% (ESI† Text S1). The obtained dye decolorizing bacterial flora was named as DDMY2.

### 2.3. Experiments on enhancement effect of tea residue

In order to investigate the enhancement effect of tea residue, eight groups were carried out and they were dye + distilled water (DD), dye + medium (DM), dye + distilled water + flora DDMY2 (DDM), dye + medium + flora DDMY2 (DMF), medium (M), dye + medium + tea residue (DMT), dye + distilled water + flora DDMY2 + tea residue (DDFT), dye + medium + flora DDMY2 + tea residue (DMFT), respectively. The inoculation of flora DDMY2 was 10% (v/v) and the culture was incubated at 37 °C under static condition. After regular intervals for 24 h, aliquots (2 mL) were collected and centrifuged at 6200 × *g* for 10 min. Subsequently, the obtained supernatant was analyzed by UV-vis spectrophotometer (IMPLEN, German). Decolorization rates were measured according to the method described by Xie *et al.*^12^

### 2.4. Decolorization assay

The capacity of decolorizing RB19 by bacterial flora DDMY2 at varying conditions was assessed. The culture media were amended/incubated with different pH values (4.0–9.0), temperatures (30–45 °C) and initial dye concentrations (50–500 mg L^−1^), respectively. Different concentrations of tea residue had also been chosen (0–6 g L^−1^) to investigate the enhancement effect. If not specified, concentration of tea residue and RB19 are respective 3 g L^−1^ and 300 mg L^−1^. The inoculation, culture conditions and the method for determination of decolorization rates were the same as those in Section 2.3.

### 2.5. Biodegradation products analysis

The culture medium containing RB19 and its decolorizing solution were harvested and centrifuged at 6200 × *g* for 10 min to obtain the supernatant. Afterwards, the supernatant was scanned through UV-vis spectrophotometry. FTIR analysis was performed to determine the changes in functional groups of dye before and after decolorization. The preparation steps are shown in ESI† Text S2. Prepared samples were measured on NICOLET 6700 spectrophotometer (Thermo, America) in the mid-IR region of 600–4000 cm^−1^ with 16-scan speed at room temperature. Besides, the biodegradation products after decolorization were also analyzed by liquid chromatography/time-of-flight/mass spectrometry (LC-TOF-MS, Agilent QTOF6520, USA). The test information is shown in ESI† Text S3.

### 2.6. High-throughput sequencing analysis

Microbial community structures of initial flora DDMY2 (not domesticated by tea residue, sample initial-DDMY2) and flora DDMY2 (domesticated by tea residue for 12 months) were investigated *via* high-throughput sequencing method reported by Xie *et al.*^12^ The specific experimental procedures are mentioned in ESI† Text S4 and all sequences had been deposited in the NCBI GenBank with accession no. SRP132025.

### 2.7. Statistical analysis

All experiments and measurements were conducted in triplicate. All data were shown as the means ± standard deviations. The Student “*t*” test or one-way analysis of variance (ANOVA) was used to evaluated significant differences of two levels of a single factor or more than two levels of a single factor, respectively. The level *p*-value ≤ 0.05 was regarded to be statistically significant and the level *p*-value ≤ 0.01 was considered to be highly significant.

## 3. Results and discussion

### 3.1. Decolorization performance of flora DDMY2

**Fig. 1:**
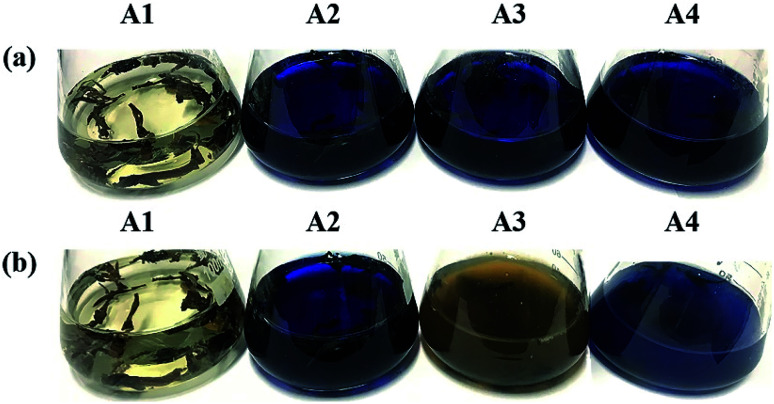
Images of 300 mg L^−1^ RB19 before (a) and after (b) decolorization by flora DDMY2 under the activation of tea residue. A1 represented medium with tea residue, A2 represented medium with tea residue and RB19, A3 represented medium with tea residue, RB19 and flora DDMY2, A4 represented medium with tea residue, RB19 and heat-inactivated flora DDMY2.

It took 150 generations (12 months) to possess the decolorizing bacterial flora DDMY2 through the method of dye gradient pressure domestication. The obtained flora DDMY2 had efficient decolorization performance of RB19 (300 mg L^−1^) and its efficiency reached up to 98% with tea residue after 48 h. From Fig. 1, it was obvious that the dark blue colored dye changed to light yellow-brown in the presence of flora DDMY2 and tea residue (group A3), speculating RB19 was decolorized which might be caused by either adsorption or degradation. Comparing group A1 (medium + tea residue) with group A2 (RB19 + medium + tea residue), both of them showed no significant color change (*p*-value = 0.241) and the color removal of group A2 after 48 h was only 2.52 ± 0.56%, indicating the adsorption of tea residue could be negligible. The group A4 with heat-inactivated flora DDMY2 (inactivated in a high-pressure steam sterilization pot at 121 °C, 0.12 MPa for 20 min), medium, tea residue and RB19 was just 6.54 ± 1.09% of decolorization after 48 h. Thus, the contribution of abiotic process (*e.g.* chemical degradation and adsorption of heat-inactivated flora DDMY2) for RB19 removal can be excluded.^13^ Summing up the above, the decolorization of RB19 was attributed to the biodegradation by flora DDMY2 with tea residue.

### 3.2. Enhancement effect of tea residue

**Fig. 2:**
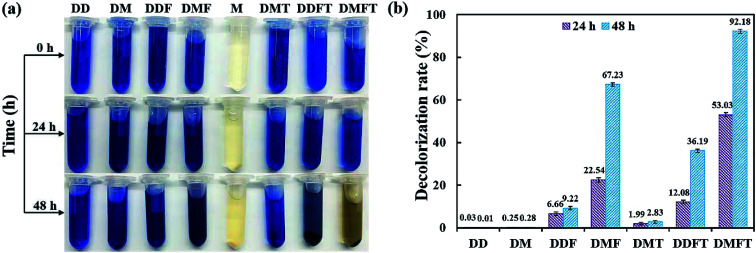
Images of enhancement effect of tea residue on RB19 decolorization by flora DDMY2 (a) and decolorization rates of tea residue towards RB19 by flora DDMY2 (b). DD: dye + distilled water; DM: dye + medium; DDF: dye + distilled water + flora DDMY2; DMF: dye + medium + flora DDMY2; M: medium; DMT: dye + medium + tea residue; DDFT: dye + distilled water + flora DDMY2 + tea residue; DMFT: dye + medium + flora DDMY2 + tea residue. Group M had no decolorization rate, so it was not included in the histogram.

The confirmatory study on the enhancement effect of tea residue towards RB19 decolorization by flora DDMY2 was carried out. From Fig. 2a, it showed that only the group DMFT (dye + medium + flora DDMY2 + tea residue) was notably decolorized from dark blue to light orange, followed by group DMF (dye + medium + flora DDMY2), which was lightly decolorized to light blue. The difference between these two groups was due to the addition of tea residue (*p*-value = 0.003). Combined with Fig. 2b, group DMFT had the highest decolorization rate of 92.18 ± 0.98% after 48 h, followed by group DMF (67.23 ± 0.82%). The 24.95% increase demonstrated tea residue played a vital role in boosting decolorization and had a strong enhancement effect. Decolorization rates of group DDF (dye + distilled water + flora DDMY2) and group DDFT (dye + distilled water + flora DDMY2 + tea residue) were 9.22 ± 0.79% and 36.19 ± 0.75%, respectively. It proved that flora DDMY2 could be also promoted by tea residue without any other nutrients (*p*-value = 0.002), but the effect was limited. Compared with group DMF (dye + medium + flora DDMY2), group DM (dye + medium) had the decolorization rate of 0.28 ± 0.07%, suggesting decolorization required the action of flora DDMY2 and it made a highly significant contribution (*p*-value < 0.01). In addition, group DMT (dye + medium + tea residue) was only with decolorization rate of 2.83 ± 0.58%, manifesting tea residue could not be used as adsorbent to remove RB19 as described in Section 3.1. This finding was different from existing research that tea residue was used as adsorbent to remove dyes.^14,15^ Based on our previous work, through single factor and orthogonal experimental analysis of common substances in tea residue, epigallocatechin gallate was deciphered to be the key active component which was responsible for the enhancement effect on the decolorization performance by activating the functional bacteria.^16^

### 3.3. Effects of physico–chemical parameters on decolorization performance of DDMY2

#### 3.3.1. Effect of pH

**Fig. 3:**
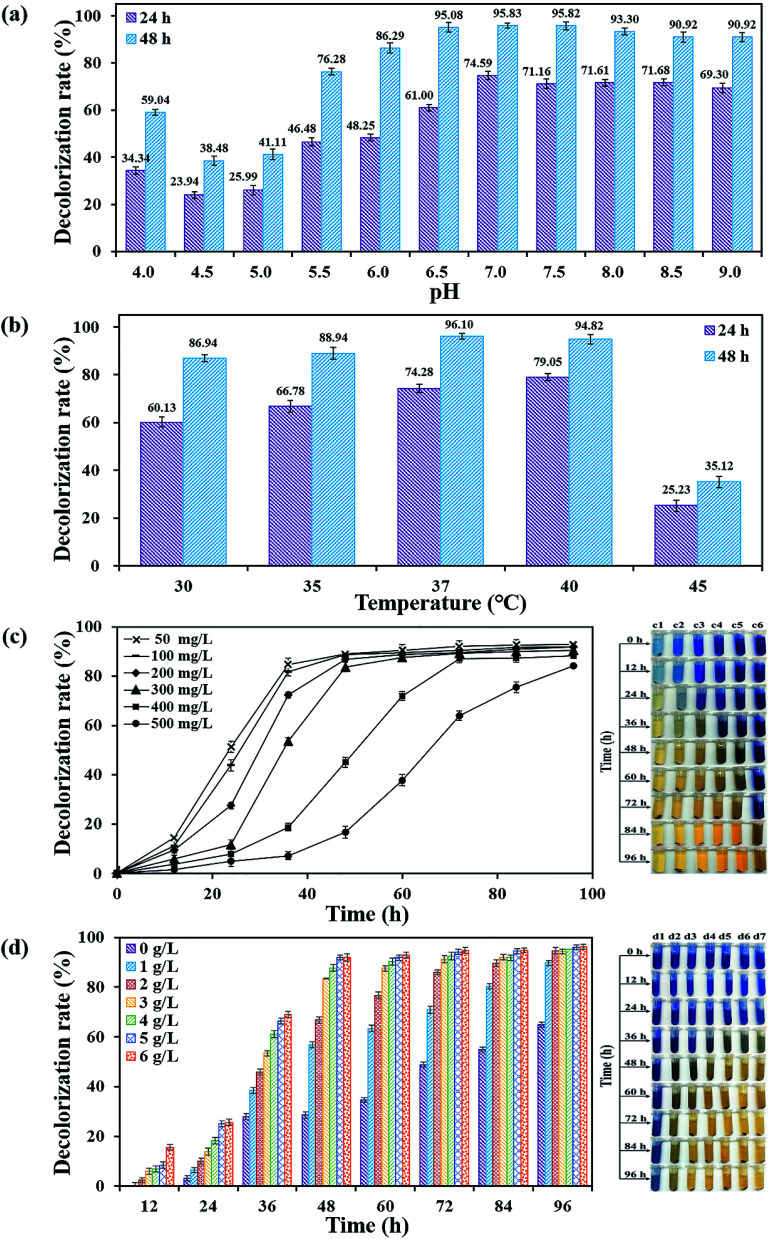
Decolorization potentiality of flora DDMY2 under the activation of tea residue at different pH values (a), temperatures (b), initial dye concentrations (c) and concentrations of tea residue (d). The right part of (c) and (d) were images of decolorization samples with different initial dye concentrations and different concentrations of tea residue. Serial numbers from c1 to c6 represented initial dye concentration 50–500 mg L^−1^, d1 to d7 represented concentrations of tea residue 0–6 g L^−1^, respectively.

Compared to optimum pH 5–7, extreme pH can severely inhibit microbial activities and even cause death.^17^ In this work, flora DDMY2 could adapt to a wide range of pH in the presence of tea residue and it showed better decolorization performance with the increase of pH value from 6.5 to 9.0 (Fig. 3a). The maximum decolorization rate was observed at pH 7.0 (95.83 ± 1.11% of decolorization rate, *p*-value < 0.01), which was similar to the result reported by Chen *et al.*^18^ When pH value was lower than 7.0 presented acidic condition, the decolorization rate rapidly decreased. In addition, flora DDMY2 also exhibited considerable decolorization effect under neutral and alkaline conditions. The pH value from neutral to weakly alkaline had already been found to have the maximum decolorization of different dyes.^19–21^ The reason might be that the neutral and weakly alkaline conditions were suitable for the growth of flora DDMY2. Moreover, it was related to the enzymatic system involved in the degradation process. For instance, some functional enzymes (*e.g.* laccase) of dye decolorizing microbes are usually stable at alkaline conditions.^22^

#### 3.3.2. Effect of temperature

Temperature is an important factor affecting the biological process of dye degradation. Under the activation of tea residue, flora DDMY2 displayed the best decolorization efficiency towards RB19 at 37 °C (96.10 ± 1.31% of decolorization rate, *p*-value < 0.01), followed by 40 °C and 35 °C (Fig. 3b). This phenomenon was coincided with the result that dye decolorization was presented at the most suitable temperature of 35 °C and 37 °C.^23^ Wang *et al.*^24^ also discovered range of 27–37 °C was the optimum decolorization temperature of reactive red by bacterial strain *Citrobacter* sp. CK3. The temperature value higher or lower than this range (37–40 °C) was found obviously reduce the decolorization capacity of flora DDMY2 in this study. For example, after 48 h, the decolorization rate only achieved 35.12 ± 2.31% at 45 °C, which decreased about 60% as compared to that at 37 °C (*p*-value = 0.002). In addition, through measuring OD_600_ and extracellular quinone reductase activity (involved in the degradation of RB19) at different temperatures, it was found that at 48 h, the biggest OD_600_ (3.24 ± 0.02) and maximum enzymatic activity (34.27 ± 1.35 μM of NADH reduced min^−1^ mg^−1^ of protein) were at 37 °C (data not shown), which was consistent with the maximum decolorization rate. Hence, it was considered that the changes in cell viability or bacterial enzymatic activity on the grounds of different temperatures caused the changes in decolorization performance towards RB19 by flora DDMY2 under the activation of tea residue.^25^

#### 3.3.3. Effect of initial dye concentration

In actual wastewater treatment process, the dye concentration is usually serious and has certain inhibitory effect on microorganisms.^26^ As a consequence, initial dye concentration was set from 50 mg L^−1^ to 500 mg L^−1^ and the decolorization effect was shown in Fig. 3c. The decolorization curves stated that, after 48 h, the decolorization performance remained stable (>80%) when RB19 was not exceeding 300 mg L^−1^ (*p*-value = 0.314). However, when the concentration was more than 400 mg L^−1^, it still reached higher decolorization efficiency but took a longer time (96 h) to adapt the pressure derived from high dye concentration.^27,28^ This suggested that flora DDMY2 had outstanding potential to decolorize high concentration of RB19 and was relatively better than other bacterial strains under the activation of tea residue.^11,29^ The phenomenon was similar to the result founded by Fu *et al.*^30^ Moreover, even if the decolorization efficiency decreased with increasing dye concentration, the actual moles of RB19 removed were increased as a function of RB19 concentration. For example, at 48 h, 88.69 ± 1.80% decolorization of 100 mg L^−1^ RB19 was equivalent to 88.69 ± 1.80 mg L^−1^ of dye removed; however, 83.73 ± 1.57% decolorization of 300 mg L^−1^ RB19 was equivalent to 251.19 ± 4.71 mg L^−1^ of dye removed. This case was also mentioned by Holkar *et al.*^31^ in the biodegradation process of RB19 by *Klebsiella* sp. C NCIM 5546.

#### 3.3.4. Effect of tea residue concentration

Different concentrations of tea residue had obvious influence on the decolorization performance by flora DDMY2. For the first 48 h, with the increase of tea residue concentration, the decolorization rate increased observably (*p*-value < 0.01, Fig. 3d). However, when tea residue concentration exceeded 3 g L^−1^, the promotion of increasing concentration was not so obvious from 48 h to 96 h (*p*-value > 0.05). The reason for this phenomenon might be: (i) the promotion for flora DDMY2 had reached the maximum limitation; (ii) many residual antimicrobial substances in tea residue accumulated and then offset the enhancement effect. From the changes of apparent color, it was observed that as tea residue concentration increased from 0 g L^−1^ to 6 g L^−1^, the color changed significantly from dark blue to light yellow. Based on these results, the optimum concentration of tea residue for activating decolorization was suggested as 3 g L^−1^ in this study.

### 3.4. Analysis of biodegradation products

**Fig. 4:**
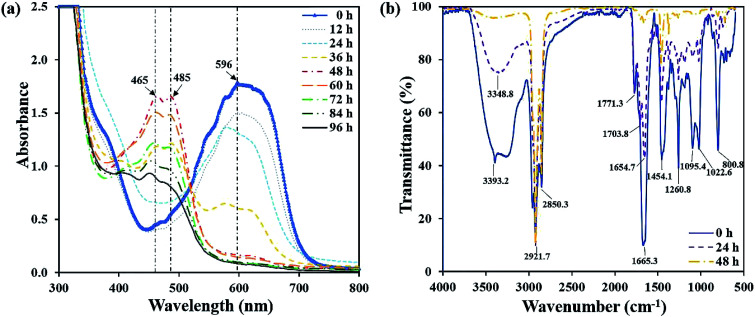
UV-vis spectra full-wavelength scanning of the decolorization process (a), and FTIR spectra at different time of the decolorization process (b).

The UV-vis spectra full-wavelength scanning results of the decolorizing solution during the decolorization process were collected. Fig. 4a showed that from 0 h to 48 h, a peak centered at 596 nm decreased, which was the characteristic absorption peak representing RB19. Especially, at 48 h, the characteristic peak intensity almost completely disappeared. Simultaneously, two new absorption peaks centered at 465 nm and 485 nm appeared and amplified gradually. Finally, they reached the highest intensity at 48 h. After another 48 h, the newly generated peaks centered at 465 nm and 485 nm began to decrease, demonstrating these intermediates were further degraded by flora DDMY2. Xu *et al.*^32^ reported that bacteria degraded dyes into intermediates which were further transformed into final products.

The FTIR spectra of RB19 differed significantly before and after decolorization, which indicated there was biodegradation in the decolorization process (Fig. 4b). The spectrum of 0 h showed the prominent absorption peak at 3393.2 cm^−1^ for stretching vibration of N–H on –NH_2_, which was the primary amino group in RB19.^33^ As decolorization proceeded, the peak 3393.2 cm^−1^ decreased significantly and disappeared completely after 48 h, indicating that the primary amino group –NH_2_ converted to secondary amino group –NH. The peaks at 2921.7 cm^−1^ and 2850.3 cm^−1^ represented the symmetry and asymmetry of C–H on alkane (–CH_2_–) stretching vibration and their intensity became higher after 48 h. The peak 1665.3 cm^−1^ was the C=O stretching vibration, which represented the vibration of the carbonyl group in the anthraquinone structure of RB19. It decreased gradually over time, indicating the anthraquinone group was degraded in this process. The peak 1454.1 cm^−1^ was the deformation vibration of C–H on –CH_2_. The peak at 1260.8 cm^−1^ represented C–N stretching vibration on the benzene ring. The peaks at 1095.4 cm^−1^ and 1022.6 cm^−1^ represented S=O stretching vibration,^34^ which came from the sulfoxide group of RB19. These decreased and absent absorption peaks revealed bonds between anthraquinone ring and benzene ring were broken and RB19 had been decomposed by flora DDMY2, thereby greatly improved the biodegradability, which was beneficial for further treatment.

The products came from flora DDMY2 decolorizing RB19 for 48 h were analyzed by LC-TOF-MS. From these results, a total of three major metabolites were identified in the positive and negative ion modes. The first product was detected in positive ion mode, presenting the peak retention time (14.629 min) and the mass-to-charge ratio (*m*/*z* 102.1200) (Fig. S1a–c†). Compared the mass spectrum with the NIST library, it was confirmed to be hexan-1-amine with a matching degree of 85.89% (Table 1), which was decomposed from RB19 by the biochemical action of flora DDMY2. The amines came from RB19 decolorization were also found by Vasconcelos *et al.*^35^ In the negative ion mode, the separation effect was obvious and there were four distinct ion peaks which were detected mainly concentrated in 9–10 min (Fig. S1d†). The peaks retention time of 10.020 min and 9.458 min (Fig. S1e and f†) corresponded to *m*/*z* 156.9900 and 194.9500, respectively (Fig. S1g and h†). The former was identified as benzenesulfonate ion with a matching degree of 90.01% and the latter was regarded as 3,6-dihydroxyphthalic acid with a matching degree of 83.50% (Table 1), which was formed by the quinone ring opening. Products of similar structures were often reported, such as dibutyl phthalate and 2,6-di-*tert*-butyl-4 methyl-phenol were detected by Castillo and Barcelo.^36^

**Table 1:** Products of RB19 after 48 h decolorization identified by LC-TOF-MS analysis

Dye	Peak retention time (min)	Mass-to-charge ratio (*m*/*z*)	Products	Chemical structure	Matching degree
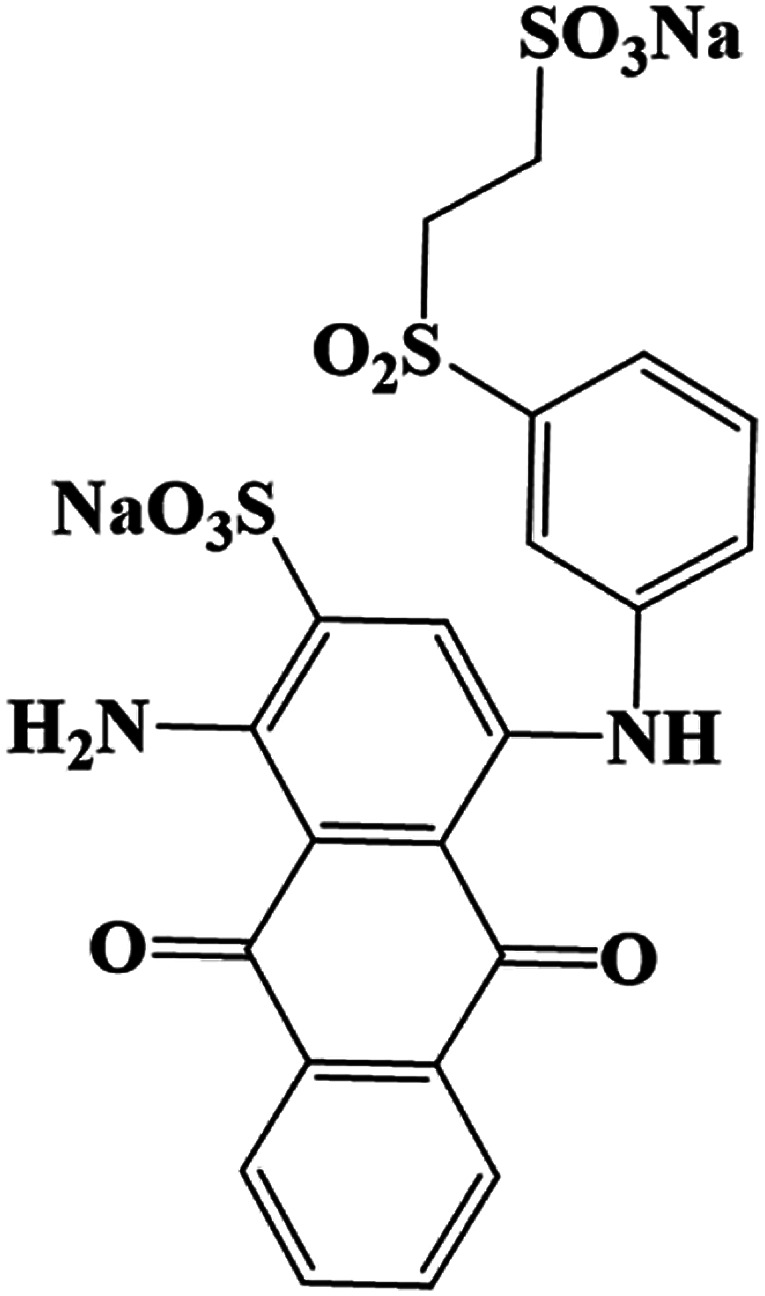	14.629	102.1200	Hexan-1-amine (C_6_H_15_N)	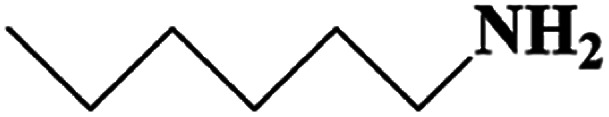	85.89%
	10.020	156.9900	Benzenesulfonic acid (C_6_H_6_O_3_S)	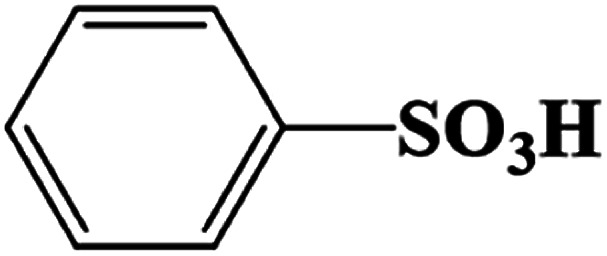	90.01%
	9.458	194.9500	3,6-Dihydroxyphthalic acid (C_8_H_6_O_6_)	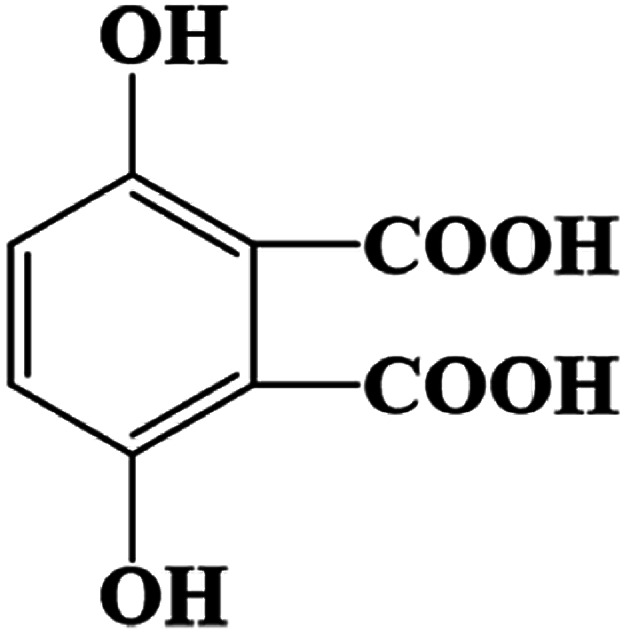	83.50%

**Fig. 5:**
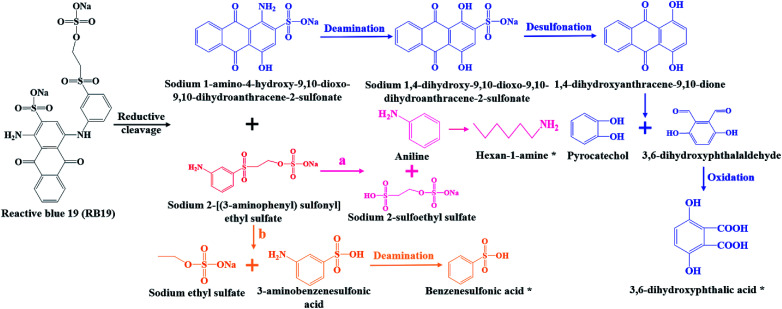
Derivation of molecular transformation mechanism of RB19 by bacterial flora DDMY2 under the activation of tea residue. (a) and (b) were two possible metabolic pathways, the symbol (*) represented the detected products.

Combined UV-Vis, FTIR and LC-TOF-MS analysis results, the chemical transformation process of RB19 by flora DDMY2 under the activation of tea residue was deduced in Fig. 5. The dye RB19 was directly hydrolyzed into sodium 1-amino-4-hydroxy-9,10-dioxo-9,10-dihydroanthracene-2-sulfonateand and sodium 2-[(3-aminophenyl) sulfonyl] ethyl sulfate. The former was determinated by Bilal *et al.*^37^ in photo-assisted catalytic degradation of RB19. The latter was also revealed by Fanchiang and Tseng^38^ in degradation of RB19. Subsequently, the amino group of sodium 1-amino-4-hydroxy-9,10-dioxo-9,10-dihydroanthracene-2-sulfonateand transformed into hydroxy group by hydrolysis or aromatic substitution to form sodium 1,4-dihydroxy-9,10-dioxo-9,10-dihydroanthracene-2-sulfonate, which was certified by McCallum *et al.*^39^ Then, the 1,4-dihydroxyanthracene-9,10-dione formed by desulfonation, which was detected by Ghazalian *et al.*^40^ in visible light photocatalytic degradation of RB19. With the process going on, the quinone ring further opened and formed pyrocatechol and 3,6-dihydroxyphthalaldehyde. The pyrocatechol was shown in the electro-Fenton process for degradation of RB19.^41^ The 3,6-dihydroxyphthalaldehyde was oxidized to 3,6-dihydroxyphthalic acid which had been detected in this study. For sodium 2-[(3-aminophenyl) sulfonyl] ethyl sulfate, it could be seen that there might be two possible metabolic pathways. In one way, sodium 2-[(3-aminophenyl) sulfonyl] ethyl sulfate was hydrolyzed into aniline and sodium 2-sulfoethyl sulfate, where aniline was found by He *et al.*^42^ in mineralization of RB19 by ozonation combined with sonolysis. The benzene ring of aniline was further opened and converted to hexan-1-amine found in this work. In the other way, sodium 2-[(3-aminophenyl) sulfonyl] ethyl sulfate might be hydrolyzed into 3-aminobenzenesulfonic acid and sodium ethyl sulfate. Subsequently, 3-aminobenzenesulfonic acid was transformed into benzenesulfonic acid by deamination, which was discovered in this research. The whole degradation pathway of RB19 by flora DDMY2 under the activation of tea residue was firstly described in this paper.

### 3.5. Microbial community structure analysis

High-throughput sequencing analysis was used to reveal the microbial community structures of sample intial-DDMY2 and sample DDMY2, which produced 40 066 and 38 685 sequence tags, respectively. Of these produced sequence tags, 34 628 reads were randomly selected for further study. In addition, a total of 24 and 33 OTUs were individually obtained from initial-DDMY2 and DDMY2 with clustering at a 3% dissimilarity level. The rarefaction curve can be used to compare the richness, uniformity or diversity of samples and indicate whether the reads of samples are reasonable. In this study, the Sobs rarefaction curves became relatively flat with the increase of sequences (Fig. S2a†), indicating the results were reasonable and exact.^43^ Based on the Shannon rarefaction curves (Fig. S2b†), sample DDMY2 had the higher community diversity.

**Fig. 6:**
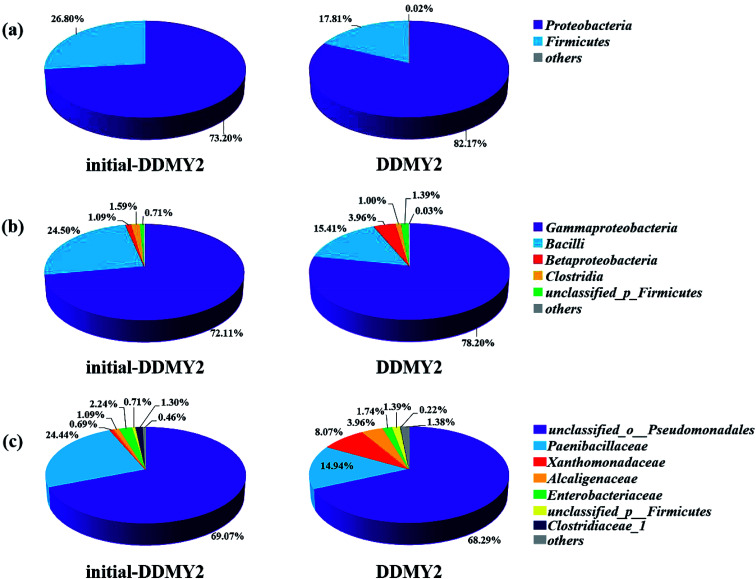
Microbial community structures of sample initial-DDMY2 and sample DDMY2 on different classification levels: phylum level (a); class level (b); family level (c), and the group *others* represented the sum of the level percentage less than 1%.

In order to further analyze the community composition of sample initial-DDMY2 and sample DDMY2 at different taxonomic levels, sequences were divided into different taxonomic classifications through RDP classifier. On classification of phylum level (Fig. 6a), two phyla *Proteobacteria* and *Firmicutes* were detected separately in two samples, which were frequently discovered in the dye biodegradation systems.^44,45^*Proteobacteria* was richer than *Firmicutes* in two samples and the proportion of *Proteobacteria* increased under the activation of tea residue, indicating *Proteobacteria* might be enriched by tea residue and played vital roles in RB19 biodegradation. On the class level (Fig. 6b), *Gammaproteobacteria* (72.11% and 78.20%, respectively) was the absolutely dominant class in sample initial-DDMY2 and sample DDMY2, followed by *Bacilli* (24.50% and 15.41%, respectively), *Betaproteobacteria* (1.09% and 3.96%, respectively), *etc. Gammaproteobacteria* was widely presented in the hydrolytic acidification process of dyeing wastewater treatment.^46^*Bacilli* and *Betaproteobacteria* were also reported to exist in simulated printing wastewater reactors.^43^ Further analysis was carried out to reveal the microbial community abundance on the family level (Fig. 6c). The *unclassified*_*o*_*Pseudomonadales* was the predominant family in initial-DDMY2 and DDMY2, which occupied 69.07% and 68.29%, respectively. The proportions were nearly no change. However, the classes with greater abundance variation in initial-DDMY2 and DDMY2 were *Paenibacillaceae* (24.44% and 14.94%, respectively) and *Xanthomonadaceae* (0.69% and 8.07%, respectively).

**Fig. 7:**
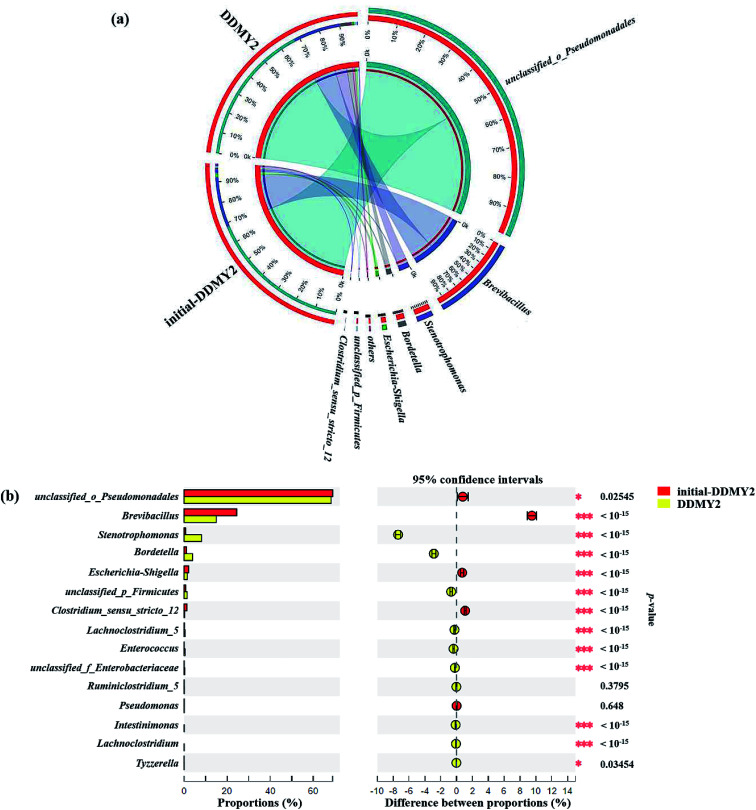
Microbial community distribution of sample initial-DDMY2 and sample DDMY2 at genus level (a), among group *others* represented the sum of the level percentage less than 1%, and the *width of the bars* from genus level indicated the relative abundance in each sample. The Fisher' exact test bar plot on genus level between sample initial-DDMY2 and sample DDMY2 (b), among the column length corresponding to the genus indicated the relative abundance in each sample, and the right part represented the *p*-value (* 0.01 < *p* ≤ 0.05; ** 0.001 < *p* ≤ 0.01; *** *p* ≤ 0.001). Significance level: 0.05. Multiple check correction: False Discovery Rate.

The Circos map showed the profiles of genus in sample initial-DDMY2 and sample DDMY2. As shown in Fig. 7a, seven genera accounting for more than 1% were detected. Among them, *unclassified*_*o*_*Pseudomonadales* made up the largest proportions (69.07% and 68.29%, respectively) of the total effective sequences in sample initial-DDMY2 and sample DDMY2, followed by *Brevibacillus* (24.44% and 14.94%, respectively), *Stenotrophomonas* (0.69% and 8.07%, respectively) and *Bordetella* (1.09% and 3.96%, respectively). It has been found that the consortium of bacteria, including *Brevibacillus*, was used to decolorize textile industry effluent.^47^ What's more, *Stenotrophomonas* had also been reported to have the ability to decolorize azo dye Xylene Fast Yellow 2G.^48^ In order to more intuitively observe the changes in the proportions of genera belonging to sample initial-DDMY2 and sample DDMY2, the Fisher' exact test bar plot was shown in Fig. 7b. Compared initial-DDMY2 with DDMY2, the percentages of *unclassified*_*o*_*Pseudomonadales*, *Brevibacillus*, *Escherichia*–*Shigella*, *Clostridium*_*sensu*_*stricto*_12 and *Pseudomonas* in sample DDMY2 decreased. The decreasing amplitudes of them were not significant (except for *Brevibacillus*), and *unclassified*_*o*_*Pseudomonadales* and *Brevibacillus* might play a certain role in decolorization because of their lager proportions. However, *Stenotrophomonas* and *Bordetella* increased remarkably (increased by 7.38% and 2.87%, respectively), which related to the enhancement effect of tea residue. The remaining eight genera only showed a slight increase. Hence, it was speculated that *unclassified*_*o*_*Pseudomonadales*, *Brevibacillus*, *Stenotrophomonas* and *Bordetella* might be mainly responsible for decolorizing RB19 by DDMY2 under activation of tea residue.

## 4. Conclusions

Bacterial flora DDMY2 possessed high decolorization performance towards RB19 under the activation of tea residue. Flora DDMY2 activated by tea residue could efficiently decolorize RB19 under conditions with wide ranges of pH values, temperatures and initial dye concentrations. Concentration of tea residue also had significant effect on decolorization performance. UV-vis, FTIR and LC-TOF-MS results indicated RB19 was degraded into small organics and the possible degradation pathway was speculated. High-throughput sequencing results revealed the changes in the community structures of initial-DDMY2 and DDMY2, of which *unclassified*_*o*_*Pseudomonadales*, *Brevibacillus*, *Stenotrophomonas* and *Bordetella* might be activated by tea residue. These findings could provide new insights of biodegradation process of anthraquinone dyes by bacterial flora, and broaden our knowledge of utilization of tea residue in the treatment of industrial dyeing wastewater.

## Conflicts of interest

There are no conflicts to declare.

## Supplementary Material

RA-009-C9RA04507D-s001
