# Supplementary material for: Highly efficient biodegradation of reactive blue 19 under the activation of tea residue by a newly screened mixed bacterial flora DDMY2
Source: RSC Adv. 2019 Aug 9;9(43):24791–801. doi: 10.1039/c9ra04507d (PMC9069888; doi:10.1039/c9ra04507d)
Supplement: RA-009-C9RA04507D-s001 [file RA-009-C9RA04507D-s001.pdf]

Electronic Supplementary Material (ESI) for RSC Advances.
This journal is © The Royal Society of Chemistry 2019

Supporting Information

Highly efficient biodegradation of reactive blue 19 under the activation of tea residue by a newly screened mixed bacterial flora DDMY2

Xuehui Xie,*[ab] Xiulin Zheng,[a] Chengzhi Yu,[a] Qingyun Zhang,[a] Yiqin Wang,[a] Junhao Cong,[a] Na Liu,[c] Zhenjiang He,[d] Bo Yang*[a] and Jianshe Liu[ab]

[a] College of Environmental Science and Engineering, Donghua University, Shanghai 201620, China; State Environmental Protection Engineering Center for Pollution Treatment and Control in Textile Industry, Donghua University, Shanghai 201620, China;

[b] Shanghai Institute of Pollution Control and Ecological Security, Shanghai 200092, P.R. China;

[c] School of Environment and Surveying Engineering, Suzhou University, Suzhou, Anhui 234000, China;

[d] School of Metallurgy and Environment, Central South University, Changsha 410083, P.R. China.

Email: xiexuehui@dhu.edu.cn, xiulinzxl@163.com, hyywsdklts@163.com, zqyfqyy@163.com, 18321195601@163.com, junhaocong@163.com, liuna900301@163.com, hzjcsu@csu.ehu.cn, yangbo@dhu.edu.cn, liujianshe@dhu.edu.cn.

* Corresponding author: Donghua University, 2999# North Renmin Road, Songjiang District, Shanghai, China. Fax: (+86)-21-67792522. E-mail: xiexuehui@dhu.edu.cn, yangbo@dhu.edu.cn.

There are 8 pages, containing 4 Texts and 2 Figures.

Text S1:

Screening of RB19 decolorizing bacterial flora DDMY2

The CM medium (90 mL) was added in a 250 mL flask, and then inoculated 10 mL activated sludge suspension with 10% (v/v). The dye concentration gradually increased in sequences of 10, 20, 30, 50, 100, 200 and 300 mg L^{-1}. The culture was incubated at 37 ℃ under static condition for 48 h. When the decolorization rate at a certain dye concentration reached up to 80% or more after 48 h, it was transferred to a new medium with higher dye concentration, which was named gradient pressure acclimation method.[1] This process was repeated until dye concentration reached to 300 mg L^{-1} and the decolorization rate was 70%, the domestication process stopped and bacterial flora was collected, named as DDMY2.

Text S2:

Sample preparation for FTIR analysis

The culture medium containing RB19 and its decolorizing solution were harvested and centrifuged at 6200 × *g* for 10 min to obtain the supernatant. Afterwards, the achieved supernatant was extracted using dichloromethane (three times with 40, 30 and 30 mL, respectively) to obtain the extraction products. Subsequently, the extraction was transferred to an eggplant-shaped flask, placed in a water bath at 30 ℃ and subjected to rotary evaporation. With the solution including extraction dried out, dichloromethane (2 mL) was used to dissolve the extraction. The new solution was concentrated to dry under nitrogen and the dried extraction was placed on the Attenuated Total Reflection instrument (Smart TR diamond).

Text S3:

Test information for LC-TOF-MS

The decolorizing culture (100 mL) after 48 h incubation was centrifuged at 6200 × *g* for 10 min to remove biomass. Subsequently, the supernatant was transferred to a 500 mL beaker and diluted. After that, two 1 kD dialysis bags were used to install 50 mL distilled water and methanol, respectively, placed in the beaker and stirred dialysis for 24 h. Finally, the solution in two dialysis bags was homogeneously mixed, 2 mL of the dialysate was taken to centrifuge at 9300 × *g* for 10 min. Subsequently, the supernatant was filtered through a 0.22 μm aqueous syringe filter and transferred to a 2 mL chromatographic vial, using LC-TOF-MS spectrometer (Agilent QTOF6520, USA) for analysis.

Chromatographic conditions: the column using Agilent zorbax 300 Extend-C18 4.6 * 150 mm, column packing particle size 3.5 μm. Binary mobile phase, phase A is ultrapure water containing 20 mM ammonium acetate and phase B is methanol containing 20 mM ammonium acetate. Phase B from 0 min (5%) → 10 min (95%) → 18 min (5%) → 20 min (5%) → 30 min (5%). The DAD detector was used to detect the substances of 205 nm, 256 nm, 280 nm, 320 nm, 360 nm, 460 nm, 480 nm, 529 nm, 596 nm and 620 nm.

Mass spectrometry conditions: sprayer pressure is 40 psig and dry gas is 350 ℃ nitrogen, 9 L min^{-1}. ESI voltage is 3500 V, capillary voltage is 170 V and cone hole voltage is 65 V. In positive and negative ion mode detections, mass spectrometry scanning range is *m/z* 50-1100. The biodegradation products were identified according to reaction time and mass spectra in the National Institute of Standards and Technology (NIST) library.

Text S4:

High-throughput sequencing

The initial flora DDMY2 (sample initial-DDMY2) and flora DDMY2 (sample DDMY2) which had been domesticated by tea residue for 12 months, were respectively obtained and centrifuged at 6200 × *g* for 10 min to collect biomass and then DNA of the biomass was extracted by E.Z.N.A.® Soil DNA Kit (Omega Bio-tek, Norcross, GA, U.S.) according to manufacturer's protocols. Subsequently, primers 515 F (5'-GTGCCAGCMGCCGCGG-3') and 907 R (5'- CCGTCAATTCMTTTRAGTTT-3') were used to amplify V4 and V5 regions of the 16S rDNA gene. The 20 μL PCR reaction mixture was composed of 4 μL 5 × FastPfu Buffer, 2 μL 2.5 mM dNTPs, 0.8 μL each of forward and reverse primer (5 μm), 0.4 μL FastPfu Polymerase and 10 ng template DNA. The PCR protocol consisted of an initial 3 min denaturation at 95 ℃, followed by 27 cycles of denaturing (95 ℃ for 30 s), annealing (55 ℃ for 30 s), extending (72 ℃ for 45 s) and completed with a final extension at 72 ℃ for 10 min. The PCR products were examined on a 2% (w/v) agarose gel, purified with the AxyPrepDNA Gel (Axygen, CA, USA) and quantified using QuantiFluor ST (Promega, USA). The purified amplicons were pooled in equimolar and paired-end sequenced (2 × 250) on an Illumina MiSeq platform according to the standard protocols.[2,3]

After sequencing, raw fastq files were demultiplexed and quality-filtered using Quantitative Insights into Microbial Ecology (QIIME). Therefore, 34628 reads had been randomly picked from the sample, the reads were grouped into operational taxonomic units (OTUs) with 3% dissimilarity cutoff using UPARSE (version 7.1 http://drive5.com/uparse/)

and chimeric sequences were identified and removed using UCHIME. The rarefaction curve was generated using Mothur program, and the representative sequences were assigned at different taxonomic levels using RDP Classifier (http://rdp.cme.msu.edu/) against the silva (SSU115) 16S rRNA database using confidence threshold of 70%.[4]

References

1 X. Xie, F. Fan, X. Yuan, N. Liu and J. Liu, *Journal of Donghua University. Natural Science Edition*, 2013, **39**, 802-813.

2 N. Liu, X. Xie, B. Yang, Q. Zhang, C. Yu, X. Zheng, L. Xu, R. Li and J. Liu, *Environ. Sci. Pollut. Res.*, 2017, **24**, 252-263.

3 X. Xie, N. Liu, J. Ping, Q. Zhang, X. Zheng and J. Liu, *Chemosphere*, 2018, **201**, 578-585.

4 K. R. Amato, C. J. Yeoman, A. Kent, N. Righini, F. Carbonero, A. Estrada, H. R. Gaskins, R. M. Stumpf, S. Yildirim, M. Torralba, M. Gillis, B. A. Wilson, K. E. Nelson, B. A. White and S. R. Leigh, *ISME J.*, 2013, **7**, 1344-1353.

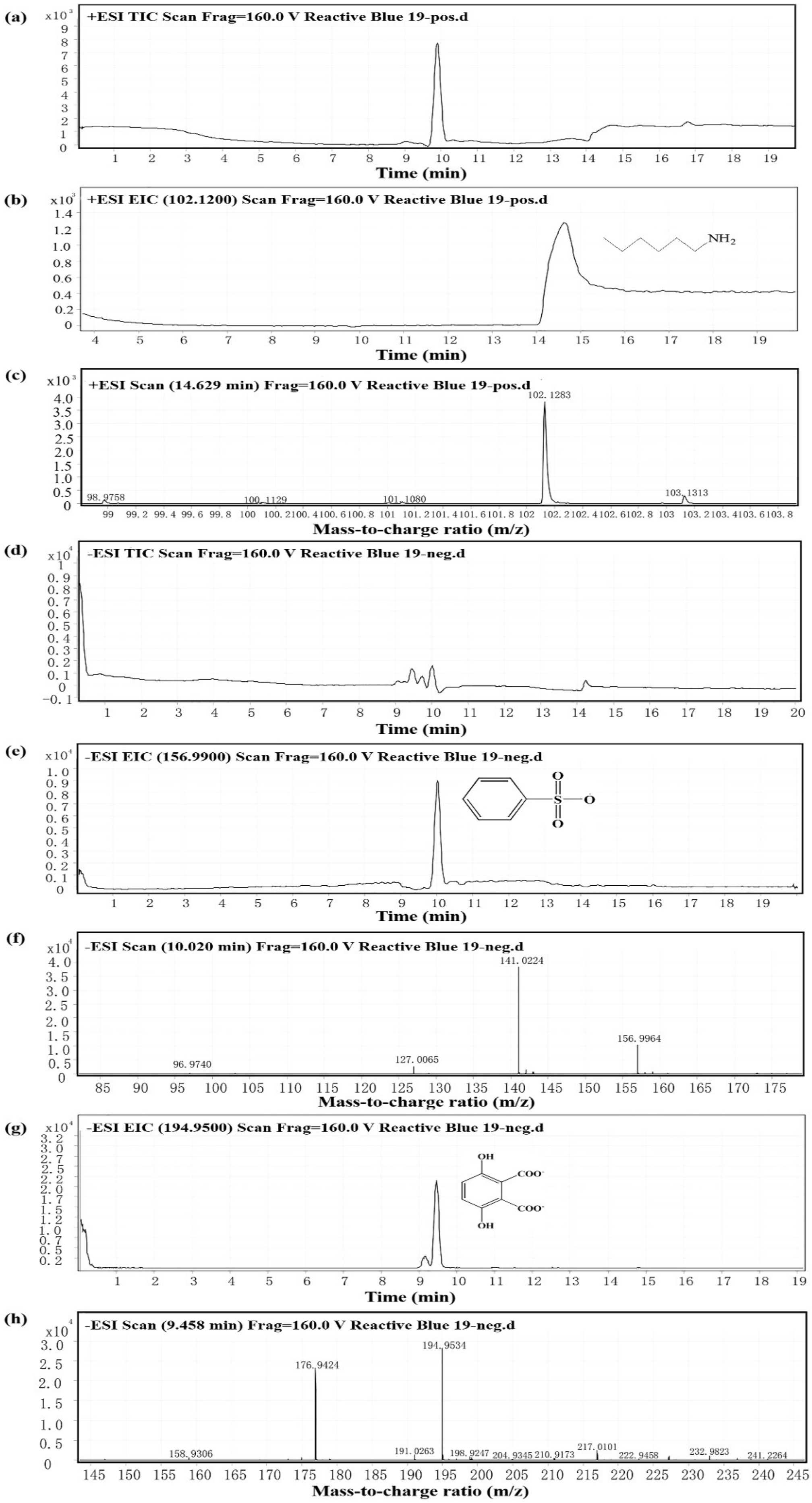
(a)
+ESI TIC Scan Frag=160.0 V Reactive Blue 19-pos.d
Time (min)
(b)
+ESI EIC (102.1200) Scan Frag=160.0 V Reactive Blue 19-pos.d
NH2
Time (min)
(c)
+ESI Scan (14.629 min) Frag=160.0 V Reactive Blue 19-pos.d
102. 1283
98. 9758
100. 1129
101. 1080
103. 1313
Mass-to-charge ratio (m/z)
(d)
-ESI TIC Scan Frag=160.0 V Reactive Blue 19-neg.d
Time (min)
(e)
-ESI EIC (156.9900) Scan Frag=160.0 V Reactive Blue 19-neg.d
Time (min)
(f)
-ESI Scan (10.020 min) Frag=160.0 V Reactive Blue 19-neg.d
141. 0224
156. 9964
127. 0065
96. 9740
Mass-to-charge ratio (m/z)
(g)
-ESI EIC (194.9500) Scan Frag=160.0 V Reactive Blue 19-neg.d
OH
COO-
COO-
OH
Time (min)
(h)
-ESI Scan (9.458 min) Frag=160.0 V Reactive Blue 19-neg.d
194. 9534
176. 9424
158. 9306
191. 0263
198. 9247
204. 9345
210. 9173
217. 0101
222. 9458
232. 9823
241. 2264
Mass-to-charge ratio (m/z)

Fig. S1 Degradation products of RB19 detected and identified by LC-TOF-MS: total ion chromatogram - positive ion mode (a); *m/z* 102.1200 ion chromatogram - positive ion mode (b); *m/z* 102.1200 ion mass spectrum - positive ion mode (c); total ion chromatogram - negative ion mode (d); *m/z* 156.9900 ion chromatogram - negative ion mode (e); *m/z* 156.9900 ion mass spectrum - negative ion mode (f); *m/z* 194.9500 ion chromatogram - negative ion mode (g); *m/z* 194.9500 ion mass spectrum - negative ion mode (h).

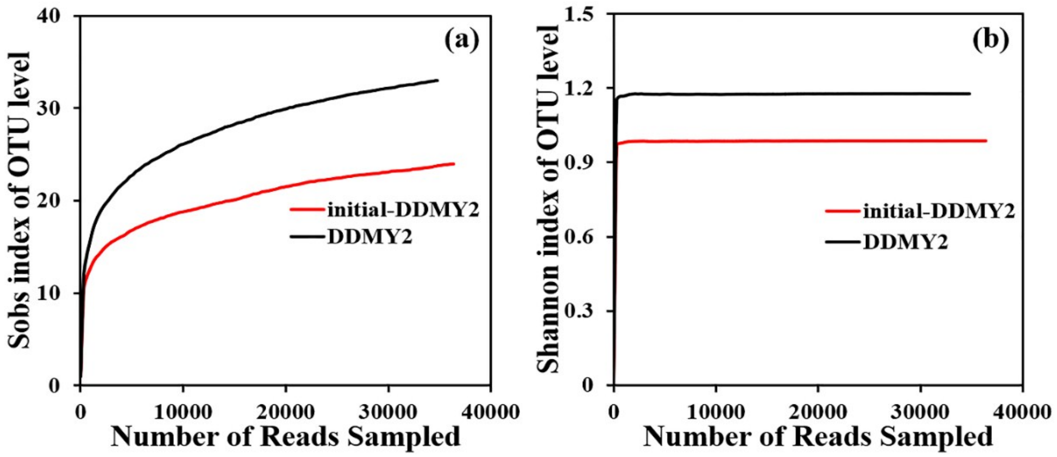

Fig. S2 Sobs (a) and Shannon (b) rarefaction curves based on the 16S rDNA gene sequencing of sample initial-DDMY2 and sample DDMY2.
